# Changes in brain function during negative emotion processing following cognitive–behavioural therapy in depressive disorders

**DOI:** 10.1192/bjp.2025.71

**Published:** 2026-04

**Authors:** Tiana Borgers, Esther Zwiky, Melissa Klug, Verena Enneking, Lukas Fisch, Lydia Klein, Laura Neutz, Elisabeth Johanna Leehr, Nils Opel, Philine König, Konrad Schöniger, Antonia Küttner, Janine Selle, Udo Dannlowski, Ronny Redlich

**Affiliations:** Institute for Translational Psychiatry, University of Münster, Germany; Department of Psychology, University of Halle, Germany; Department of Psychiatry and Psychotherapy, University Hospital Jena, Germany; German Center for Mental Health (DZPG), Halle–Jena–Magdeburg, Germany; Center for Intervention and Research on Adaptive and Maladaptive Brain Circuits Underlying Mental Health (C-I-R-C), Halle–Jena–Magdeburg, Germany

**Keywords:** Cognitive–behavioural therapy, depression, functional magnetic resonance imaging, emotion processing, limbic system

## Abstract

**Background:**

Cognitive–behavioural therapy (CBT) is a first-line treatment for depressive disorders, but research on its neurobiological mechanisms is limited. Given the heterogeneity in CBT response, investigating the neurobiological effects of CBT may improve response prediction and outcomes.

**Aims:**

To examine brain functional changes during negative emotion processing following naturalistic CBT.

**Method:**

In this case-control study, 59 patients with depressive disorders were investigated before and after 20 CBT sessions using a negative-emotion-processing paradigm during functional magnetic resonance imaging, clinical interviews and depressive symptom questionnaires. Healthy controls (*n* = 60) were also assessed twice within an equivalent time interval. Patients were classified into subgroups based on changes in diagnosis according to DSM-IV criteria (*n* = 40 responders, *n* = 19 non-responders). Brain activity changes were examined using group × time analysis of variance for limbic areas, and at the whole-brain level.

**Results:**

Analyses yielded a significant group × time interaction in the hippocampus (*P* family-wise error [*P*_FWE_] = 0.022, *η*_*P*_^2^ = 0.101), and a significant main effect of time in the dorsal anterior cingulate cortex (*P*_FWE_ = 0.043, *η*_*P*_² = 0.098), resulting from activity decreases following CBT (*P*_FWE_ ≤ 0.024, *η*_*P*_² ≤ 0.233), with no changes in healthy controls. Hippocampal activity decreases were driven by responders (*P*_FWE_ ≤ 0.020, *η*_*P*_² ≤ 0.260) and correlated with symptom improvement (*r* = 0.293, *P* = 0.024). Responders exhibited higher pre-treatment hippocampal activity (*P*_FWE_ = 0.017, *η*_*P*_² = 0.189).

**Conclusions:**

Following CBT, reduced activity in emotion-processing regions was observed in patients with depressive disorders, with hippocampal activity decreases linked to treatment response. This suggests successful CBT could correct biased emotion processing, potentially by altering activity in key areas of emotion processing. Hippocampal activity may function as a predictive marker of CBT response.

Cognitive models of depression^1^ describe low mood and behavioural withdrawal as consequences of distorted emotion processing and maladaptive thinking. Empirical evidence highlights selective attention to, recall of and difficulty disengaging from negative information as key features of these distortions.^2–6^ Cognitive–behavioural therapy (CBT) addresses these biases by, for example, promoting changes in biased attention.^7^ Although CBT is a first-line treatment for depressive disorders,^8^ a notable proportion of patients do not respond sufficiently^9^ and research on its underlying mechanisms remains limited. Investigating the neurobiological effects of CBT may offer insights into mechanisms and predictors to improve this therapy.

Functional neuroimaging (fMRI) studies in depressive disorders suggest an imbalance in frontolimbic activity during negative emotion processing, with hyperactivity in the limbic system and hypoactivity in prefrontal regions such as the dorsal anterior cingulate cortex (dACC).^10,11^ To date, few longitudinal CBT fMRI studies have been conducted,^12–15^ yielding a rather inconsistent pattern concerning the reversibility of these activity changes during negative emotion processing. While some studies show activity decreases in the hippocampus–amygdala complex^12^ and increases in the ACC^12,14^ and prefrontal areas^13,14^ following CBT, other evidence suggests increased hippocampal activity in response to negative stimuli following CBT.^13^ Additionally, one study^15^ reported no significant changes in brain activity related to negative emotion processing following CBT. Furthermore, the extent to which these neural changes correlate with clinical symptom improvement remains unclear.^14,15^ Previous studies were probably limited by small sample size (*n* ≤ 23) and a lack of statistical comparisons with control groups (except for ref. ^12^), making it difficult to determine whether the observed effects were due to the treatment itself or general fluctuations. Additionally, predicting treatment response appears crucial due to the heterogeneity in outcome. Previous CBT fMRI studies suggest lower pre-treatment dACC activity during negative emotion processing as a potential predictor of CBT response,^12,13^ but results are based on symptom questionnaires prone to weekly fluctuations. Only one study has applied machine learning,^16^ identifying pre-treatment dACC and frontal activity as potential classifiers for CBT response. However, because the sample size in this study was rather small (*N* = 16), further predictive models at the individual patient level are needed for clinical translation. Thus, the present study aimed to examine the effect of CBT on brain function and its link to treatment response using a longitudinal naturalistic design, based on a supraliminal emotion-processing task in patients with depressive disorder before and after approximately 20 naturalistic CBT sessions. We focused on the hippocampus, amygdala and dACC, given their relevance for emotion processing. First, we hypothesised a decrease in amygdala and hippocampus activity and an increase in dACC activity to negative stimuli following CBT in patients (objective a). Furthermore, we investigated whether these activity changes were related to response (objective b). We further expected pre-treatment activity differences between responders and non-responders (objective c). Additionally, we exploratively applied machine learning analyses to test predictive models, aiming to identify reliable neural predictors of CBT response at the individual level and to support personalised treatment decisions.

## Method

### Participants and study design

In this longitudinal naturalistic study, participants were assessed by fMRI, the structured clinical interview for DSM-IV (SCID-I),^17^ as well as by the Hamilton Depression Rating Scale (HDRS)^18^ and Beck Depression Inventory (BDI).^19^ Additionally, the course of illness was obtained. For patients with depressive disorders, assessments took place before the start (baseline, *t*_0_) and following approximately 20 sessions of naturalistic CBT (follow-up, *t*_1_; Supplement 1). The healthy control (HC) group was assessed at equivalent time points (time [*T*], _*T*0–*T*1_, in months: *M*_patients_ = 7.81, s.d._patients_ = 2.42; *M*_HC_ = 7.70, s.d._HC_ = 1.83; *P* = 0.773). Patients were recruited from the psychotherapeutic outpatient unit of the University of Muenster (Psychotherapie-Ambulanz), while healthy controls were recruited via public notices and newspaper announcements. Both groups were part of the ongoing Prevention and Intervention Neuroimaging Cohort study, with data collection for these groups conducted between August 2017 and September 2022. The authors assert that all procedures contributing to this work comply with the ethical standards of the relevant national and institutional committees on human experimentation, and with the Helsinki Declaration of 197 as revised in 2013. All procedures involving human participants were approved by the Ethics Committee of the University of Münster (amendment nos 2016-173-f-S and 2020-205-f-S). Written informed consent was obtained from all participants.

The final study sample consisted of *n* = 59 patients with a depressive disorder and *n* = 60 healthy controls (for exclusion criteria and sample selection, see Supplement 2 and Supplementary Fig. 1). Sample characteristics can be found in Table 1. The patient group was further divided into two subgroups (Table 2) based on the change in diagnosis from *t*_0_ to *t*_1_ according to DSM-IV.^17^ Patients who transitioned from acute to partial or full remission, or from partial to full remission, between *t*_0_ and *t*_1_, were considered as responders (*n* = 40). Patients who had suffered from an ongoing acute or partially remitted depressive disorder since *t*_0_ were considered as non-responders (*n* = 19). Detailed information on prior treatments in patients is presented in Supplementary Table 1.

**Table 1:** Sociodemographic and clinical characteristics of the sample

Characteristics	Patient group *n* = 59	Healthy control group *n* = 60	*P*-value^a^
	Mean (s.d.)	Mean (s.d.)	
Sociodemographic characteristics			
Age at *t*_0_, years	27.12 (7.48)	38.30 (17.18)	<0.001
Sex (m/f)^b^	26/33	20/40	0.229
Time between *t*_0_ and *t*_1_ measurement, months	7.81 (2.42)	7.70 (1.83)	0.773
Time between *t*_0_ and treatment start, days^c^	17.84 (13.20)	–	–
Symptom severity			
HDRS *t*_0_	12.85 (6.10)	1.47 (2.09)	<0.001
HDRS *t*_1_	7.03 (5.77)	0.90 (1.41)	<0.001
BDI *t*_0_^d^	21.12 (7.80)	1.88 (2.17)	<0.001
BDI *t*_1_^e^	11.94 (8.51)	1.32 (2.22)	<0.001
Clinical characteristics at baseline			
Diagnosis (MDD/dysthymia/adjustment disorder with depressed mood), no. of patients	56/1/2	–	–
Disease progression of MDD before *t*_0_ (first episode/recurrent)	21/35	–	–
Duration of current depressive episode, months^f^	19.58 (33.95)	–	–
No. of depressive episodes before *t*_0_^g^	2.68 (2.45)	–	–
Cumulative duration of depressive episodes before *t*_0_, months^h^	33.898 (41.86)	–	–
Cumulative duration of in-patient treatment before *t*_0_, months	0.85 (1.73)	–	–
Age of onset, years	21.01 (7.86)	–	–
Remission status (no/partial/full remission), no. of patients	44/15/0	–	–
Clinical characteristics at follow-up			
No. of CBT sessions between *t*_0_ and *t*_1_	20.83 (3.92)	–	–
Remission status (no/partial/full remission), no. of patients	11/31/17	–	–
Cumulative duration of in-patient treatment between *t*_0_ and *t*_1_, months	0.16 (0.79)	–	–
Comorbidity			
Acute comorbidity (no/yes), no. of patients at *t*_0_	31/28	–	–
Acute comorbidity (no/yes), no. of patients at *t*_1_	39/20	–	–
Psychopharmacological treatment^i^			
Medication load index at *t*_0_	0.53 (0.88)	–	–
Medication load index at *t*_1_	0.59 (0.95)	–	–

HDRS, Hamilton Depression Rating Scale; BDI, Beck Depression Inventory; MDD, major depressive disorder; CBT, cognitive–behavioural therapy.

a . *P*-values were obtained using the unpaired two-tailed *t*-test except where noted.

b . *P*-values were obtained using the *χ* ^2^-test.

c . Information was missing for *n* = 21 in the patient group.

d . Information was missing for *n* = 1 in the healthy controls group.

e . Information was missing for *n* = 2 in the patient group and *n* = 1 in the healthy controls group.

f . Information was missing for *n* = 6 in the patient group.

g . Information was missing for *n* = 2 in the patient group.

h . Information was missing for *n* = 3 in the patient group.

i . For information on medication load index, see Supplement 10.

**Table 2:** Sociodemographic and clinical characteristics of responders versus non-responders

Characteristics	Responders *n* = 40	Non-responders *n* = 19	*P*-value^a^
	Mean (s.d.)	Mean (s.d.)	
Sociodemographic characteristics			
Age at *t*_0_, years	27.75 (7.60)	25.79 (7.24)	0.351
Sex, m/f^b^	17/23	9/10	0.725
Symptom severity			
HDRS *t*_0_	12.87 (6.15)	12.79 (6.17)	0.961
HDRS *t*_1_	5.40 (4.68)	10.46 (6.44)	0.005
BDI *t*_0_	20.50 (7.25)	22.42 (8.93)	0.382
BDI *t*_1_^c^	9.97 (6.62)	16.23 (10.59)	0.030
Clinical characteristics at baseline			
Diagnosis (MDD/dysthymia/adjustment disorder with depressed mood), no. of patients^b^	37/1/2	19/0/0	0.472
Disease progression of MDD before *t*_0_ (first episode/recurrent)^b^	15/22	6/13	0.379
Subtype of MDD at *t*_0_ (melancholic/atypical/seasonal/no subtype)^b^,^d^	16/6/1/9	7/2/0/2	0.861
Duration of current depressive episode, months^e^	17.17 (34.86)	24.68 (32.36)	0.458
Number of depressive episodes before *t*_0_	2.66 (2.18)	2.74 (2.98)	0.910
Cumulative duration of depressive episodes before *t*_0_, months^f^	32.80 (47.08)	36.04 (30.21)	0.787
Cumulative duration of in-patient treatment before *t*_0_, months	0.62 (1.19)	1.33 (2.49)	0.250
Age at onset, years	21.50 (7.79)	20.00 (8.12)	0.498
Remission status (no/partial/full remission), no. of patients^b^	33/7/0	11/8/0	0.043
Clinical characteristics at follow-up			
No. of CBT sessions between *t*_0_ and *t*_1_	20.40 (4.37)	21.74 (2.62)	0.224
Remission status (no/partial/full remission), no. of patients at *t*_1_^b^	0/23/17	11/8/0	<0.001
Cumulative duration of in-patient treatment between *t*_0_ and *t*_1_, months	0.04 (.24)	0.41 (1.34)	0.246
Comorbidity			
Acute comorbidity (no/yes), no. of patients at *t*_0_^b^	18/22	13/6	0.092
Acute comorbidity (no/yes), no. of patients at *t*_1_^b^	26/14	13/6	0.795
Psychopharmacological treatment^g^			
Medication load index at *t*_0_	0.55 (0.82)	0.47 (1.02)	0.758
Medication load index at *t*_1_	0.43 (0.68)	0.95 (1.31)	0.116

HDRS, Hamilton Depression Rating Scale; BDI, Beck Depression Inventory; MDD, major depressive disorder; CBT, cognitive–behavioural therapy.

a . *P*-values were obtained using the unpaired two-tailed *t*-test except where noted.

b . *P*-values were obtained using the *χ* ^2^-test.

c . Information was missing for *n* = 1 in the responder group.

d . Subtypes that did not occur in our sample (psychotic, catatonic, postpartal) were not listed; *n* = 8 responders and *n* = 8 non-responders were partially remitted at *t*_0_ and therefore did not exhibit any subtype.

e . Information was missing for *n* = 4 in the responder group and *n* = 2 in the non-responder group.

f . Information was missing for *n* = 3 in the responder group.

g . For information on medication load index, see Supplement 10.

### fMRI paradigm, data acquisition and preprocessing

For details on the paradigm, data acquisition and preprocessing, as well as on first-level analyses of fMRI data, see Supplements 3–5 and Supplementary Fig. 2. Briefly, for the fMRI paradigm, a negative emotional face-processing task was implemented. This consisted of four blocks of a face-processing task using photographs of faces expressing fear or anger from the Ekman and Friesen^20^ stimulus set, and five blocks of a sensorimotor control task featuring geometric figures in the shape of circles or ellipses. T2* functional data were obtained using a 3 Tesla scanner (Prisma, Siemens, Erlangen, Germany) at *t*_0_ and *t*_1_, and preprocessed using statistical parametric mapping software (SPM8, Wellcome Department of Cognitive Neurology, London, UK; http://www.fil.ion.ucl.ac.uk/spm). A contrast image was generated for each participant in their individual first-level analysis (faces versus shapes), comparing activation while viewing negative faces versus shapes.

### Statistical analyses

Clinical and demographic data were analysed using SPSS Statistics version 29.0, IBM Corporation. To assess the impact of CBT on depressive symptom severity, paired *t*-tests were conducted to compare HDRS and BDI scores at *t*_0_ and *t*_1_ within patients. Additionally, 2 × 2 repeated-measures analyses of variance (ANOVAs) were performed on HDRS and BDI scores, with response (responders versus non-responders) as the between-subjects factor and time (*t*_0_ *v*. *t*_1_) as the within-subjects factor. Parametric tests were used in these analyses to enhance comparability with prior treatment fMRI studies. As a robustness check, statistical analyses were additionally performed using non-parametric tests.

#### Functional activity analysis

The fMRI data were analysed using statistical parametric mapping software (SPM12, v6685, Wellcome Department of Cognitive Neurology, London, UK; http://www.fil.ion.ucl.ac.uk/sp), with age and sex as covariates of no interest. For each of the following analyses, region of interest (ROI) analyses of the bilateral hippocampus, bilateral amygdala and bilateral dACC were performed separately. ROIs were created using the Wake Forest University PickAtlas,^21^ with hippocampus and amygdala ROI defined according to the AAL-atlas^22^ and dACC ROI defined as BA 32^23^ (dilated by 1 mm). Whole-brain analyses were also performed, reporting results with a cluster size threshold of *k* ≥ 100. Significance thresholds for multiple testing were obtained at the cluster level using threshold-free cluster enhancement (TFCE) implemented in the TFCE toolbox, version 174 (http://dbm.neuro.uni-jena.de/tfce). For all following analyses, a conservative family-wise error (FWE)-corrected threshold of *P* < 0.05 was established by performing 10 000 permutations per test.

To test the hypothesis regarding CBT-related activity changes (objective a), a 2 × 2 ANOVA, with group (patients versus healthy controls) as a between-subjects factor and time (*t*_0_ *v. t*_1_) as a within-subjects factor, was conducted. To investigate whether pre-treatment activity and activity changes are associated with response (objectives b and c), a subsequent 2 × 2 ANOVA was performed within the patient group, with response (responders versus non-responders) as a between-subjects factor. Post hoc paired *t*-tests in each group followed significant main effects of time and interaction effects. Group differences in activity between patients and healthy controls at *t*_0_ and *t*_1_ were tested via two-sample *t*-tests. Additionally, a multivariate machine learning approach, based on Winter et al.,^24^ was applied to distinguish responders from non-responders using pre-treatment ROI and whole-brain activity. For this approach, classification algorithms including support vector machines, random forests, logistic regression, *k*-nearest neighbours, Gaussian naive Bayes and boosting classifiers were trained; detailed information is provided in Supplement 6. To explore whether CBT-related activity changes are linked to symptom changes within patients (objective b), Spearman’s correlations were computed between significant activity changes and ΔHDRS/ΔBDI scores. Functional activity data from significant clusters identified in 2 × 2 ANOVA (patients versus healthy controls) at *t*_0_ and *t*_1_ were extracted. Percentage change scores were calculated for brain activity, HDRS and BDI (Δvariable = (variable_*t*1_ – variable_*t*0_)/variable_*t*0_ × 100) using SPSS Statistics. Higher scores reflect increased activity or symptoms.

## Results

### Clinical effects of CBT

HDRS and BDI scores significantly decreased in patients from *t*_0_ to *t*_1_ (HDRS: *M*_Diff_ = 5.816, 95% confidence interval [CI] [4.099, 7.533], *P* < 0.001, partial *η* ^2^ [*η*_*p*_^2^] = 0.442, 95% CI [0.2476, 0.5784]; BDI: *M*_Diff_ = 9.266, 95% CI [6.952, 11.580], *P* < 0.001, *η*_*p*_^2^ = 0.535, 95% CI [0.3461, 0.6534]). Based on our response criteria, 67.8% (*n* = 40) of patients were classified as responders and 32.2% (*n* = 19) as non-responders. There were no significant differences between responders and non-responders with regard to sociodemographic and clinical variables, with the exception of BDI and HDRS scores at *t*_1_ and remission status at both time points (Table 2). A 2 × 2 ANOVA (responders versus non-responders, *t*_0_ *v*. *t*_1_) on HDRS and BDI scores showed that the significant HDRS decrease was driven by responders (*M*_DIFF_ = 7.473, *P* < 0.001, *η*_*p*_^2^ = 0.566, 95% CI [0.3396, 0.6932]), while both groups exhibited a reduction in BDI scores (responders: *M*_DIFF_ = 10.621, *P* < 0.001, *η*_*p*_^2^ = 0.569, 95% CI [0.3396, 0.6964]; non-responders: *M*_DIFF_ = 6.331, *P* < 0.001, *η*_*p*_^2^ = 0.506, 95% CI [0.1362, 0.6924]; Supplement 7). The results remained valid following robustness checks with non-parametric tests (Supplement 8).

### Effects of CBT on negative emotion processing (objective a)

**Fig. 1:**
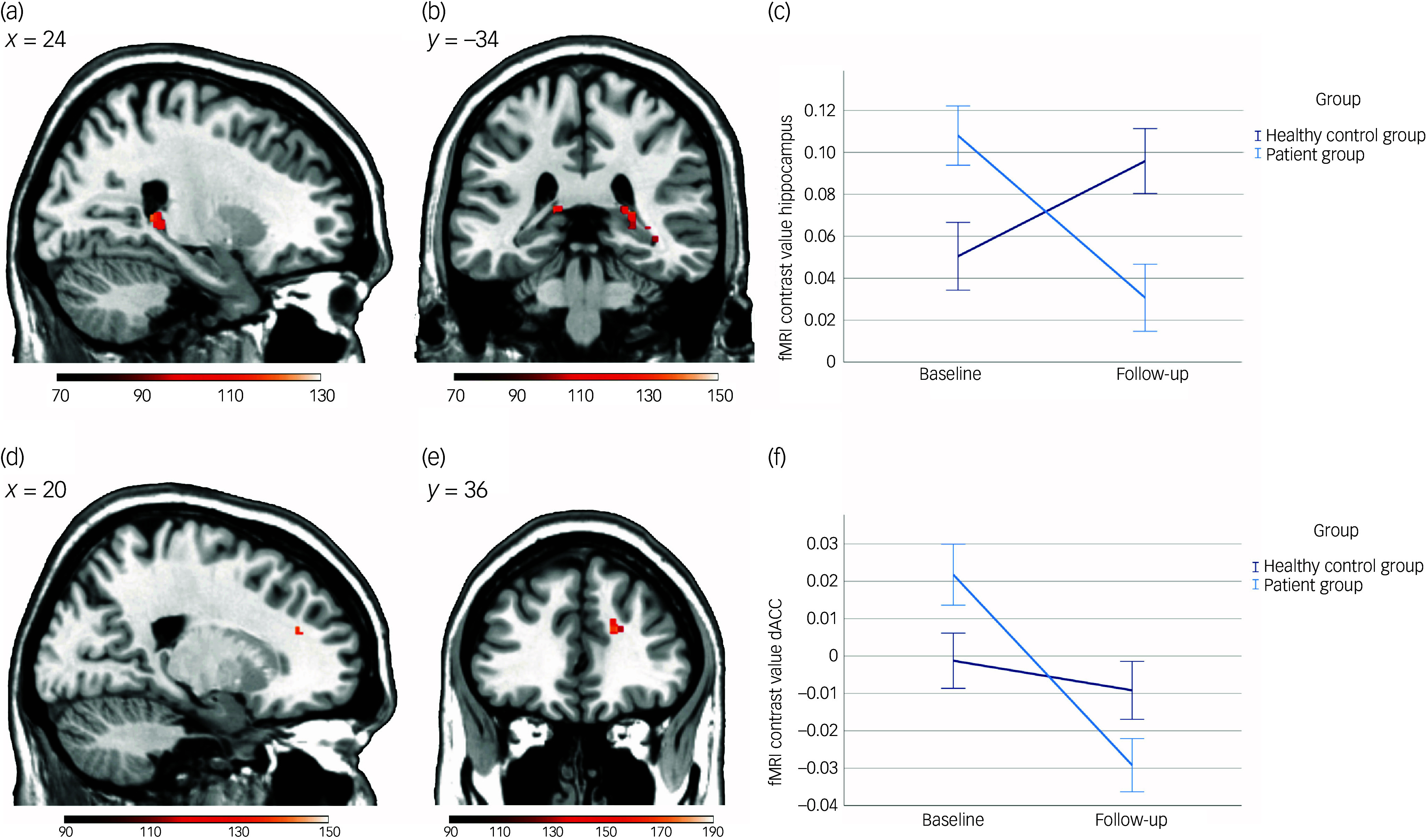
Effects of cognitive–behavioural therapy on functional activity in emotion-processing areas. (a) Visualisation of significant cluster (right: *x* = 24, *y* = −40, *z* = 6, TFCE_(232)_ = 120.33, *T*_(232)_ = 4.10, *k* = 26, *P*_FWE_ = 0.022, *η*_*p*_^*2*^ = 0.101) of the hippocampus region of interest (ROI) analysis for the group × time interaction effect (one-tailed) on a Montreal Neurological Institute (MNI) template, and (b) driven by activity decreases in the patient group from baseline to follow-up (left: *x* = −16, *y* = −34, *z* = 10, TFCE_(232)_ = 127.32, *T*_(232)_ = 4.48, *k* = 9, *P*_FWE_ = 0.024, *η*_*p*_^2^ = 0.204; right: *x* = 26, *y* = −34, *z* = 8, TFCE_(232)_ = 130.48, *T*_(232)_ = 3.93, *k* = 34, *P*_FWE_ = 0.022, *η*_*p*_^2^ = 0.185. (a,b), Clusters significant at *P*_TFCE-FWE_ < 0.05. Scale bars indicate TFCE values. (c) Plot depicting hippocampal activity at baseline and follow-up for the healthy control and patient groups during the study interval. Functional magnetic resonance imaging (fMRI) contrast values were calculated using the eigenvariate function of the significant right cluster from the hippocampus ROI analysis of the group × time interaction (one-tailed). This function extracts the first eigenvariate from the significant cluster through singular value decomposition of the time series across all voxels within the cluster. Because all second-level analyses were based on the contrast between each individual’s face and shape (defined in the first-level analysis), the plot represents typical responses for faces versus shapes in this cluster by yielding a vector of fMRI contrast values for each subject and time point. (d) Visualisation of significant cluster (right: *x* = 20, *y* = 36, *z* = 22, TFCE_(232)_ = 134.22, *T*_(232)_ = 3.94, *k* = 5, *P*_FWE_ = 0.043, *η*_*p*_^2^ = 0.098) of the dorsal anterior cingulate cortex (dACC) ROI analysis for the main effect of time (one-tailed) on a MNI template, and (e) driven by activity decreases in the patient group from baseline to follow-up (right: *x* = 20, *y* = 36, *z* = 22, TFCE_(232)_ = 174.60, *T*_(232)_ = 4.94, *k* = 11, *P*_FWE_ = 0.022, *η*_*p*_^2^ = 0.245). (d,e) Clusters significant at *P*_TFCE-FWE_ < 0.05. Scale bars indicate TFCE values. (f) Plot depicting dACC activity at baseline and follow-up for the healthy control and patient groups during the study interval. fMRI contrast values were computed by extracting the first eigenvariate of the significant right cluster resulting from the dACC ROI analysis of the main effect of time (one-tailed; for details, see (c)). *P*_FWE_, *P* family-wise error; *T*, test statistic (*t*-value); TFCE, threshold-free cluster enhancement.

There was a significant group (patients versus healthy controls) × time (*t*_0_ *v*. *t*_1_) interaction within the right hippocampus (*P*_FWE_ = 0.022, *η*_*p*_^2^ = 0.101, 95% CI [0.0210, 0.2100]; Fig. 1). The observed interaction resulted from significant activity decreases within patients (left: *P*_FWE_ = 0.024, *η*_*p*_^2^ = 0.204, 95% CI [0.0489, 0.3686]; right: *P*_FWE_ = 0.022, *η*_*p*_^2^ = 0.185, 95% CI [0.0383, 0.3497]), whereas there was no significant change in hippocampal activity within healthy controls between *t*_0_ and *t*_1_ (*P*_FWE_ = 0.420). Furthermore, a main effect of time was identified in the right hippocampus (*P*_FWE_ =0.028, *η*_*p*_^2^ = 0.124, 95% CI [0.0334, 0.2372]). Moreover, a main effect of time was observed in the right dACC (*P*_FWE_ = 0.043, *η*_*p*_^2^ = 0.098, 95% CI [0.0195, 0.2063]), marked by a significant decrease in dACC activity within patients (*P*_FWE_ = 0.022, *η*_*p*_^2^ = 0.233, 95% CI [0.0669, 0.3965]; Fig. 1), while no significant activity changes were evident in healthy controls (*P*_FWE_ = 0.120). However, no significant interaction effect was detected within the dACC (*P*_FWE_ = 0.173), and there was no significant interaction effect or main effect of time in the amygdala (both *P*_FWE_ ≥ 0.198). Supplementary Table 2 provides detailed information on the obtained results. Exploratory whole-brain analyses revealed a significant main effect of time (all *P*_FWE_ ≤ 0.040) resulting from significant activity decreases from *t*_0_ to *t*_1_ in clusters encompassing only the precuneus, middle temporal gyrus and hippocampus in patients (all *P*_FWE_ ≤ 0.033; Supplementary Table 3). There was no significant change within healthy controls (*P* = 0.159). Notably, no significant interaction effect was identified (*P*_FWE_ ≥ 0.120).

### Association of activity changes with response (objective b)

Even if the subsequent analysis of responders versus non-responders showed only a nominally significant response × time interaction within the hippocampus (*P*_FWE_ = 0.068), post hoc *t*-tests revealed that responders showed a decrease in bilateral hippocampal activity during negative emotion processing between *t*_0_ and *t*_1_ (left: *P*_FWE_ = 0.020, *η*_*p*_^2^ = 0.204, 95% CI [0.0269, 0.3999]; right: *P*_FWE_ = 0.005, *η*_*p*_^2^ = 0.260, 95% CI [0.0556, 0.4509]; Supplementary Table 2). There was no significant change in hippocampal activity in non-responders following CBT (*P*_FWE_ = 0.220). The main effect of time was not statistically significant (*P*_FWE_ = 0.087). Within the right dACC, analysis of responders versus non-responders showed a significant main effect of time (*P*_FWE_ = 0.018, *η*_*p*_^2^ = 0.219, 95% CI [0.0584, 0.3839]; Supplementary Table 2). Post hoc tests, however, demonstrated no significant dACC activity changes in either responders (*P*_FWE_ = 0.179) or non-responders (*P*_FWE_ = 0.092), and no significant interaction effect was observed within the dACC (*P*_FWE_ = 0.276). Additionally, there was no significant interaction effect or main effect of time in the amygdala (both *P*_FWE_ ≥ 0.416), or at whole-brain level in clusters *k* ≥ 100 (*P*_FWE_ ≥ 0.256).

Viewed dimensionally, reductions in left hippocampal activity within patients from *t*_0_ to *t*_1_ were associated with ΔHDRS (left: *r* = 0.293, *P* = 0.024; right: *P* = 0.935). There was no significant correlation between reductions in bilateral hippocampal activity and BDI score changes (both *P* ≥ 0.114). Reductions in right dACC activity within patients from *t*_0_ to *t*_1_ were not significantly associated with overall depressive symptom improvement (HDRS: *P* = 0.759; BDI: *P* = 0.648).

### Cross-sectional group differences and response prediction (objective c)

Cross-sectional analyses revealed no significant differences in brain activity during negative emotion processing between patients and healthy controls at either *t*_0_ or *t*_1_ across the hippocampus (both *P*_FWE_ ≥ 0.232), amygdala (both *P*_FWE_ ≥ 0.125), dACC (both *P*_FWE_ ≥ 0.424) or at whole-brain level (both *P*_FWE_ ≥ 0.221).

Concerning pre-treatment differences between the responder and non-responder groups, the subsequent analysis of responders versus non-responders revealed that the former had higher pre-treatment activity within the right hippocampus during negative emotion processing compared with non-responders (*P*_FWE_ = 0.017, *η*_*p*_^2^ = 0.189, 95% CI [0.0397, 0.3554]; Supplementary Table 2). However, no significant pre-treatment differences were observed between responders and non-responders in either the dACC (*P*_FWE_ = 0.371) or amygdala (*P*_FWE_ = 0.267; Supplementary Table 2). Similarly, no significant differences were observed between both response groups and healthy controls (all *P*_FWE_ ≥ 0.347; Supplement 9). Additionally, no pre-treatment differences in whole-brain activity were identified between responders and non-responders (*P*_FWE_ ≥ 0.264). In our machine learning analyses, the best hyperparameter configurations were achieved with random forest classification (Supplementary Table 4). Across tested brain regions, the highest mean balanced accuracy was found for pre-treatment dACC activity (60.4%). For detailed results, see Supplementary Table 5 and Supplementary Fig. 3.

### Robustness checks

Robustness checks (Supplement 10 and Supplementary Tables 6 and 7) were performed to validate the main findings, including adjustments for non-linear age effects and clinical characteristics in patients (baseline depressive symptom severity, number of depressive episodes before *t*_0_, remission status at *t*_0_, acute comorbidity and medication intake). Overall, robustness checks confirmed a consistent pattern of results.

## Discussion

The present study investigated changes in functional activity during negative emotion processing following naturalistic CBT in patients with a depressive disorder. Our results revealed that activity in emotion-processing areas (hippocampus and dACC) was decreased following CBT in patients and that hippocampal activity changes were related to CBT response. Furthermore, responders showed higher pre-treatment hippocampal activity to negative stimuli in our standard univariate analyses. Notably, these findings largely persisted after controlling for non-linear age effects and clinical characteristics.

### Effects of CBT on negative emotion processing and association with response

Consistent with objective a and one previous study,^12^ our results showed a significant interaction in the right hippocampus, driven by decreased hippocampal activity during negative emotion processing in patients following naturalistic CBT, with no changes in healthy controls. The hippocampus often shows hyperactivity in depressive disorders during emotion processing.^11^ In our study, the significant cluster was primarily located in the posterior hippocampus, an area linked to cognitive and memory processes.^25^ This suggests that the observed activity decrease may indirectly support improved emotion processing, because previous research indicates that reduced posterior hippocampal activity helps individuals with depression manage negative affect by limiting the reinstatement of mood-congruent negative memories.^26^ Importantly, hippocampal activity reductions were observed only in CBT responders, potentially suggesting that such changes may occur primarily following successful treatment. Moreover, these decreases correlated positively with greater symptom reductions as measured by HDRS but not by BDI (objective b). One explanation is the methodological variance between a clinician-rated interview and a self-report scale. HDRS may be better aligned with DSM-IV criteria, capturing psychopathological elements more apparent to external raters and thereby enhancing its capacity to detect clinically relevant changes, whereas BDI may also be influenced by maladaptive personality traits.^27,28^ Additionally, HDRS encompasses a broader range of symptom dimensions, including somatic aspects, while BDI primarily focuses on cognitive-affective symptoms.^29,30^ Consequently, hippocampal activity may be more closely linked to symptom domains as assessed by HDRS, suggesting that its reduction may not solely reflect improved emotion processing. Future research should incorporate targeted behavioural experiments to objectively evaluate emotion-processing deficits and treatment effects. Building on our findings, it remains uncertain whether hippocampal activity decreases are central to the antidepressant mechanisms of naturalistic CBT. Although healthy controls showed no significant activity changes over time, suggesting that the observed decreases in patients may not merely reflect the passage of time, these reductions could still represent a general correlate or consequence of symptom improvement rather than a CBT-specific effect. Alternatively, they may arise from common therapeutic factors such as the therapeutic alliance. To clarify these influences, future research would benefit from the use of standardised CBT protocols and the inclusion of waitlist and active treatment control groups. Notably, hippocampal activity decreases are not unique to CBT. Similar reductions have been observed with antidepressants, although the majority of studies found no association with treatment response.^31,32^ In line with this, the lack of a significant response × time interaction in our study warrants caution in interpreting hippocampal activity decreases as strictly response-dependent CBT effects.

Contrary to objective a, our analyses yielded a significant main effect of time, reflecting significant dACC activity decreases during negative emotion processing following naturalistic CBT in patients, while no significant changes were observed in healthy controls. Involvement of the (dorsal) ACC has been implicated in both top-down emotional regulation^33^ and – within the salience network – rumination and self-referential processing.^34^ The observed reduction in dACC activity following CBT may therefore either indicate improved efficiency in emotion regulation – for example, through a reduced need for cognitive control and limbic inhibition,^35^ given the concurrent hippocampal activity decrease – or improvements in maladaptive processes such as rumination. There was no significant association between dACC activity decreases and symptom changes, nor any differential changes in dACC activity between responders and non-responders, despite the main effect of time (objective b). This suggests that dACC activity alterations may be unrelated to symptom changes, although our analysis focused on overall symptoms rather than specific domains such as rumination, which could explain the lack of association. The specificity of dACC decreases during negative emotion processing to CBT remains unclear. While psychotherapies generally show reduced dACC activity,^36^ electroconvulsive therapy (ECT)^37^ and antidepressants^32^ often exhibit ACC activity increases. However, evidence is mixed, with some studies reporting opposing effects following CBT^12,14^ and antidepressants.^31^ Further research should systematically compare different treatments to disentangle the effects of time, remission, treatment modality and common therapeutic factors on dACC activity, while also carefully considering ACC subregion variations.

Surprisingly, no significant amygdala activity changes were observed following naturalistic CBT, either between patients and healthy controls (objective a) or between responders and non-responders (objective b). This contrasts with studies showing reduced amygdala activity following treatments such as CBT,^12^ antidepressants^31^ and ECT.^38^ Our sample included patients with milder symptoms, such as adjustment disorder and partially remitted major depressive order, which may have limited the scope for observable amygdala changes. Additionally, detecting significant effects in a small region like the amygdala with a modest sample size remains challenging. A brief discussion of the whole-brain results is provided in Supplement 11.

### Cross-sectional group differences and response prediction

Contrary to previous studies,^10,11^ we found no significant pre-treatment differences in amygdala, hippocampus or dACC activity between patients and healthy controls. Given the proposed links between depression severity and the extent of brain activity alterations,^39^ the inclusion of outpatients with varying symptom levels may have obscured more pronounced group differences. Future studies should investigate activity differences based on symptom severity to clarify their influence on brain alterations. Our still relatively small sample size for cross-sectional analyses also probably limited the statistical power. Nevertheless, patients showed descriptively higher pre-treatment and lower post-treatment hippocampal and dACC activity compared with healthy controls, aligning with a previous study suggesting normalisation of limbic hyperactivity to the level of healthy controls following CBT.^12^ Although descriptive activity differences hint that individuals with depression may require lower-than-average activity in these emotion-processing regions, caution is warranted due to the lack of significant findings. Consequently, our results do not clearly support the reversibility of depression-related activity alterations.

Concerning pre-treatment predictors of CBT response, responders showed elevated pre-treatment hippocampal activity compared with non-responders, despite no differences at post-treatment or in amygdala and dACC activity (objective c). This suggests that the potential pathophysiological differences that may underlie the observed hippocampal activity decrease in responders following CBT, and also explains the absence of treatment response observed in non-responders. However, another study has linked pre-treatment hippocampal hypoactivity during emotion processing with CBT response,^15^ complicating the role of hippocampal hyperactivity as a predictive marker. The lack of pre-treatment dACC activity differences between responders and non-responders could hint at a response-independent effect of dACC activity decreases. It may also be explained by either the limited statistical power, the phenotypic variability of our sample or the use of an emotion-processing rather than a regulation paradigm.^40^ Understanding why some patients respond while others do not remains an open and critical question, particularly because responders and non-responders in our sample did not significantly differ in regard to prior illness course, baseline depressive symptom severity, comorbidities, medication use or baseline brain activity relative to healthy controls. These findings imply that non-response cannot be fully explained by these factors alone, and point to the potential role of neurobiological differences. Indeed, the group-level distinction in pre-treatment hippocampal activity underscores depression’s neurobiological heterogeneity. However, our exploratory machine learning analyses indicate that functional imaging data are not yet reliable for predicting individual CBT response, with classification accuracy not exceeding 61%. This is in line with recent research^24^ raising questions about the clinical utility of brain imaging markers for response prediction. Larger samples and careful consideration of clinical heterogeneity may be needed to identify robust predictors of treatment outcome. Ultimately, pinpointing reliable neural markers could enable tailored interventions, such as more intensive CBT, alternative therapies or adjusted treatment durations.^41^ Meanwhile, factors beyond the scope of this study, such as environmental influences (e.g. adverse life events, social support) or genetic predispositions, may also contribute to non-response, warranting further investigation to refine personalised treatment strategies.

### Strengths and limitations

Our study comprehensively examined activity changes during negative emotion processing and their association with CBT response in patients with depressive disorders compared with healthy controls, while also exploring pre-treatment neural markers using group statistics and a machine learning approach. The naturalistic study design provides high external validity. Unlike previous studies, we defined CBT response based on SCID-I-validated changes in clinical diagnosis, offering a more reliable measure than symptom rating scales by capturing significant suffering and functional impairment, being less susceptible to weekly fluctuations and reflecting clinical progression throughout CBT. However, some limitations must be acknowledged. First, the naturalistic study design limited control over CBT interventions because sessions were not standardised, although psychotherapists followed established manuals and national care guidelines for unipolar depression.^8^ Second, the inclusion of patients with different depressive diagnoses adds heterogeneity, possibly explaining the absence of significant cross-sectional differences between patients and healthy controls and limiting the scope of our interpretations. However, this diversity and the application of naturalistic CBT provide higher external validity and a more realistic portrayal of those seeking CBT for depression in our healthcare system. Third, our paradigm raises uncertainty about whether the findings reflect emotion processing of facial stimuli or face processing in general. Future research should include a neutral face or stimulus-absent condition. Despite this, our paradigm is well established for exploring negative emotion processing.^37,42^ Additionally, reliability constraints of paradigm-based fMRI and partial volume effects, particularly in regions close to grey–white matter boundaries, may impact result accuracy (e.g. the clusters in Fig. 1). Additional large-sample longitudinal studies are needed to validate our findings, especially given that p*η* ^2^ can overestimate effect size in small samples.

Following naturalistic CBT in patients with depressive disorders, reductions in both hippocampal and dACC activity were observed during negative emotion processing. The identified association between hippocampal activity decreases and response suggests that these changes may occur following only successful CBT, potentially reflecting improvements in biased emotion processing. Univariate analyses showed that responders and non-responders differed in pre-treatment hippocampal activity, although machine learning approaches did not support this finding. In contrast, dACC activity reductions do not seem to be linked to treatment response. Overall, these results provide insights into how naturalistic CBT may influence the neural circuitry underlying negative emotion processing in depressive disorders.

## Supporting information

Borgers et al. supplementary material 1 Borgers et al. supplementary material

Borgers et al. supplementary material 2 Borgers et al. supplementary material

## Data Availability

The data that support the findings of this study are available from the corresponding author, R.R., upon reasonable request.

## Supplementary material

The supplementary material can be found at https://doi.org/10.1192/bjp.2025.71

## Analytic code availability

The analytic code is available from the corresponding author, R.R., upon reasonable request.

## Research material availability

The research material is available from the corresponding author, R.R., upon reasonable request.
