# Supplementary material for: Changes in brain function during negative emotion processing following cognitive–behavioural therapy in depressive disorders
Source: Br J Psychiatry. 2025 May 7;228(4):324–31. doi: 10.1192/bjp.2025.71 (PMC13051210; doi:10.1192/bjp.2025.71)
Supplement: Borgers et al. supplementary material 1 — Borgers et al. supplementary material [file S0007125025000716sup001.docx]

**Supplementary Material**

[Supplement 1: Cognitive-behavioral therapy 2](#_Toc187749808)

[Supplement 2: Exclusion criteria and sample selection 2](#_Toc187749809)

[Exclusion criteria 2](#_Toc187749810)

[Sample selection 3](#_Toc187749811)

[Supplement 3: FMRI data acquisition and preprocessing 3](#_Toc187749812)

[FMRI data acquisition 3](#_Toc187749813)

[Preprocessing 4](#_Toc187749814)

[Supplement 4: Paradigm 4](#_Toc187749815)

[Supplement 5: First-level analyses 5](#_Toc187749816)

[Supplement 6: Exploratory machine learning approach 5](#_Toc187749817)

[Supplement 7: Clinical effects in relation to response 6](#_Toc187749818)

[Supplement 8: Robustness checks using non-parametric tests for analyses of clinical characteristics 6](#_Toc187749819)

[Supplement 9: Baseline activity differences between response groups and healthy controls 7](#_Toc187749820)

[Supplement 10: Robustness checks of functional activity analyses 7](#_Toc187749821)

[Methods 7](#_Toc187749822)

[Results 8](#_Toc187749823)

[Supplement 11: Effect of CBT on whole-brain activity 9](#_Toc187749824)

[Supplementary Tables 10](#_Toc187749825)

[Table S1. Details on prior treatments in the patient group 10](#_Toc187749826)

[Table S2. Results of the group x time ANOVAs in all three regions of interest 12](#_Toc187749827)

[Table S3. Results of the main effect of time at whole-brain level 14](#_Toc187749828)

[Table S4. Best hyperparameter configurations of the Random Forest Classifier 15](#_Toc187749829)

[Table S5. Detailed results of the classification accuracy based on the ROI and whole-brain results (mean and standard deviation) 16](#_Toc187749830)

[Table S6. Results of the group x time ANOVAs in all three regions of interest with age^2^ or HDRS score at baseline as additional covariates (including age and sex) 17](#_Toc187749831)

[Table S7. Results of the response x time ANOVAs in all three regions of interest with medication load index, acute comorbidity (no/ yes), remission status at baseline (no/ partial/ full remission) and number of depressive episodes before baseline, on top of age and sex, as covariates. 20](#_Toc187749832)

[Supplementary Figures 21](#_Toc187749833)

[Figure S1. Flow diagram visualizing the participant dropout process 21](#_Toc187749834)

[Figure S2. Example trial of fMRI paradigm. 22](#_Toc187749835)

[Figure S3. Classification results for responders versus non-responders at baseline 23](#_Toc187749836)

[References 24](#_Toc187749837)

# Supplement 1: Cognitive-behavioral therapy

Patients underwent 20 sessions of naturalistic individual CBT (M=20.83, SD=3.92; range: 9-30) in an outpatient setting, administered by psychotherapists in training – psychologists with completed university degrees in psychology, currently undergoing training to become licensed psychological psychotherapists specialized in cognitive-behavioral therapy. In this outpatient unit, therapies were supervised every two weeks by licensed CBT psychotherapists. Due to the naturalistic study design, the sessions were not standardized. However, supervision ensured that psychotherapists in training generally adhered to established manuals for depressive disorders and complied with the highest standards outlined in the national care guidelines for unipolar depression (1). Therefore, all therapies included core interventions such as psychoeducation, behavioral activation and cognitive restructuring (2,3). Sessions lasted 50 minutes and were scheduled approximately once per week. However, specific session durations and frequencies were not systematically recorded, as participants were externally recruited from a psychotherapy unit. Overall, the therapeutic process followed a naturalistic course, limiting the availability of detailed information on the specific content of each session.

# Supplement 2: Exclusion criteria and sample selection

## Exclusion criteria

Exclusion criteria for all participants were any neurological abnormalities, chronic medical diseases, benzodiazepine intake at study time or MRI contraindications. At t_0_, all patients fulfilled DSM-IV criteria for a primary diagnosis of an acute or partially remitted major depressive disorder (MDD), acute dysthymia or acute adjustment disorder with depressed mood which was verified with the structured clinical interview for DSM-IV (SCID-I) (4). Further exclusion criteria for patients were a diagnosis of a bipolar disorder, a psychotic disorder or an acute substance dependence. At t_0_, all patients were either on the waiting list or in the trial phase for CBT (Time between t_0_ and treatment start in days: M_patients_=17.84, SD_patients_=13.20) and subsequently received approximately 20 sessions of naturalistic CBT (excluding the trial phase) between t_0_ and t_1_. A prerequisite for inclusion was that patients scored above the cut-off (≥10) and healthy controls (HCs) scored below the cut-off (<10) for clinically remarkable depressive symptoms (5) on the BDI (6) at t_0_. Further exclusion criterion for HC was the presence of any life-time diagnosis of a mental disorder according to the SCID-I (4).

## Sample selection

The study sample was derived from the Prevention and Intervention Neuroimaging Cohort (PINC), initially including 129 patients with a depressive disorder and 119 HCs with baseline data. Subsequently, 79 participants were excluded for lacking follow-up assessment (t1) due to various reasons: 7 patients began psychotherapeutic treatments other than CBT (psychodynamic psychotherapy) after baseline (confirmed via telephone interview), 8 HCs met criteria for a mental disorder at baseline, 3 HCs had somatic conditions contraindicating MRI or other assessments in the PINC study, 39 participants were unreachable or uninterested in follow-up (n= 22 patients, n = 17 HCs), and 22 participants had not completed follow-up assessments by the start of data analysis (n = 4 patients, n = 18 HCs). Third, 19 patients with a depressive disorder who did not receive CBT (or any other psychotherapeutic treatment) between baseline and follow-up were excluded, while 12 participants (n = 8 patients, n = 4 HCs) were excluded due to unavailable fMRI images at baseline, follow-up, or both. None of the remaining brain functional images required exclusion for excessive head movement (>3 mm/3°). From the remaining 69 HCs and 69 patients, further exclusions were made: 5 HCs who developed a diagnosis during the study interval, 3 patients with fully remitted major depressive disorder or partially remitted adjustment disorder at baseline, 2 patients who underwent more than 30 CBT sessions, and 5 patients scoring below the BDI cut-off and 4 HCs scoring above the BDI cut-off at baseline (see exclusion criteria). This process yielded a final sample of 59 patients with depressive disorders and 60 HCs. For a flow diagram visualizing the participant dropout process, see **Figure S1**.

For the final study sample, complete fMRI data were available for analyses. While there were some missing values in the clinical interview data and the BDI questionnaire, these are noted in **Table 1** and **Table 2**. No specific method such as imputation was applied to handle this missing data. For the correlation analyses, participants with missing BDI values were excluded from these specific analyses. In contrast, analyses involving the HDRS or responder groups were based on complete data for the HDRS, diagnosis and remission status.

# Supplement 3: FMRI data acquisition and preprocessing

## FMRI data acquisition

Stimuli were presented at the back of the scanner (Sharp XG-PC10XE with additional HF shielding; Osaka, Japan), while participants lay supine in the MRI scanner with a response box in their right hand, and their head stabilized using a vacuum head cushion. T2* functional data were acquired at a 3 Tesla scanner (Prisma, Siemens, Erlangen, Germany) using a single-shot echoplanar sequence with parameters chosen to minimize distortion in the region of interest, maintain an adequate signal-to-noise ratio (S/N) and T2* sensitivity: 33 slices, matrix 64 x 64, resolution 3.3 × 3.3 × 3.8 mm; repetition time = 2.0 s, echo time = 29 ms, flip angle = 90°. The slices were obtained in an ascending order, phase encoding direction: anterior >> posterior. Additionally, slices were tilted 20° from the anterior and posterior commissure line to reduce dropout artifacts in the orbitofrontal and mediotemporal regions.

## Preprocessing

The functional imaging data were preprocessed using statistical parametric mapping software (SPM8, Welcome Department of Cognitive Neurology, London, UK; http://www.fil.ion.ucl.ac.uk/spm). This involved realignment using six rigid-body transformations determined for each image, unwarping and spatial normalization of each participant's functional images to the Montreal Neurological Institute International Consortium (MNI) for Brain Mapping template. The images were smoothed using a Gaussian kernel with a full-width at half-maximum (FWHM) of 6mm.

# Supplement 4: Paradigm

Functional magnetic resonance imaging (fMRI) was used to investigate the neural correlates associated with negative emotional face processing using a supraliminal paradigm. The fMRI paradigm is an adapted version of the face-matching task by Hariri et al. (7). The task comprised of four blocks of a face-processing task, alternating with five blocks of a sensorimotor control task. During each block of the face-processing task, participants were presented trials with a trio of faces (all expressing either fear or anger) from the Ekman and Friesen stimulus set (8), and were instructed to match one of two faces at the bottom of the screen that matched the target face at the top via keystroke. The sensorimotor control task showed trials with a trio of geometric shapes (circles and ellipses) in each block, following the matching procedure of the face-processing task. Each face processing block consisted of six face trios, which were balanced for emotion (as well as for sex) making it three trials with angry faces and three trials with fearful faces presented in two different orders. Each sensorimotor control block consisted of six shape trios presented in a random order. Face-processing trials lasted for four seconds with a variable inter-stimulus interval (2-6 sec), while each sensorimotor control trial lasted for four seconds with a fixed inter-stimulus interval of two seconds. The total duration of the paradigm was 390 seconds, consisting of 24 face-processing trials and 30 sensorimotor control trials. Prior to each block, an instruction asking participants to match either faces or shapes was given for two seconds. An example trial can be found in **Figure S2**.

# Supplement 5: First-level analyses

The start and duration of the experimental conditions, involving faces and shapes, were modeled with a canonical hemodynamic response function using a general linear model. The model was also adjusted for serial correlations, and a high-pass filter with a cutoff of 128s was used to eliminate low-frequency noise. For each participant, one contrast image was created in their individual first-level analysis (faces > shapes), comparing the activation patterns while viewing negative faces versus shapes.

# Supplement 6: Exploratory machine learning approach

ML analyses were conducted on whole brain level and in all three ROIs (*objective c*). The PHOTON toolbox, a tool developed within our research group ((9); <https://www.photon-ai.com>) served as the instrumental framework for these analyses. The ML approach of the study was implemented using Python 3.9.16, along with scikit-learn version 0.24.2, photonai version 2.1.0, and photonai_neuro version 0.2.0. To construct our ML pipelines, we used previously established methodologies from Winter et al. (10). This approach not only ensured a robust foundation for our analyses but also facilitated a seamless integration of cutting-edge techniques into our investigation. One ML pipeline was comprised of a series of sequential data transformation steps and a concluding classification algorithm. The data transformation steps encompassed imputation of missing data, feature normalization, selection of a subset of univariate features with the highest effect size, and a principal component analysis (PCA) aimed at reducing the dimensionality of the brain data. Following these transformations, a classification algorithm was trained for predicting response groups at baseline, encompassing support vector machines, random forests, logistic regression, k-nearest neighbour, Gaussian naive Bayes, and boosting classifiers. Due to computational reasons, we deviated from Winter et al. (10) in the cross-validation process: A nested cross-validation scheme was implemented with 5 inner validation and 5 outer test splits, serving to optimize hyperparameters and evaluate the final generalizability of the model. For detailed information, see Winter et al. (10). Balanced accuracy served as the performance metric for the models, providing a balance between sensitivity and specificity to maintain a baseline random value of 50%, regardless of imbalances in the number of samples among classified groups. Mean accuracies were reported based on 5 evaluations of the test set.

# Supplement 7: Clinical effects in relation to response

A significant response (responders vs. non-responders) x time (t_0_ vs. t_1_) interaction was observed for HDRS scores (F_(1, 57)_=8.920, p=.004, η_p_^2^=.135, 95.%-CI[.0147, .2984]), driven by a significant decrease in HDRS scores in responders (M_DIFF_=7.473, p<.001, η_p_^2^=.566, 95.%-CI[.3396, .6932]) but not in non-responders (M_DIFF_=2.329, p=.062, η_p_^2^=.181, 95.%-CI[.0000, .4482]). For BDI scores, no significant response x time interaction was found (F_(1, 55)_=3.092, p=.084, η_p_^2^=.053, 95.%-CI[.0000, .1969]), but a significant main effect of time (F_(1, 55)_=48.270, p<.001, η_p_^2^=.467, 95.%-CI[.2687, .6015]) indicating a decrease in BDI scores in both groups (Responders: M_DIFF_=10.621, p<.001. η_p_^2^=.569, 95.%-CI[.3396, .6964]; Non-responders: M_DIFF_=6.331, p<.001, η_p_^2^=.506, 95.%-CI[.1362, .6924]).

# Supplement 8: Robustness checks using non-parametric tests for analyses of clinical characteristics

The Wilcoxon signed-rank test indicated a significant decrease in HDRS and BDI scores within the patient group (HDRS: z=-4.86, p<.001; BDI: z=-5.39, p<.001) from t_0_ to t_1_. Furthermore, the Mann-Whitney U-test revealed significant differences between the patient and HC groups in HDRS scores (Baseline: U=71.50, Z=-9.11, p<.001; Follow-Up: U=418.50, Z=-7.32, p<.001) and BDI scores (Baseline: U=0.00, Z=--9.41, p<.001; Follow-Up: U=310.50, Z=-7.74, p<.001) at both time points. When comparing responders and non-responders using the same non-parametric test, significant differences were observed in HDRS and BDI scores at t_1_ (HDRS: U=203.50, Z=-2.87, p=.004; BDI: U=210.50, Z=-2.42, p=.015), whereas no significant response group differences were found at t_0_ (HDRS: U=377.50, Z=-.04, p=.971; BDI: U=341.00, Z=-.63, p=.532). The Friedman test indicated a significant reduction in HDRS scores from t_0_ to t_1_ for both responders (χ^2^_(1)_=18.69, p<.001) and non-responders (χ^2^_(1)_=6.37, p=.012). Similarly, for BDI scores, the Friedman test revealed a significant decrease from t_0_ to t_1_ in both groups (Responders: χ^2^_(1)_=25.97, p<.001; Non-responders: χ^2^_(1)_=5.56, p=.018).

# Supplement 9: Baseline activity differences between response groups and healthy controls

There was no significant baseline activity difference between the responder and HC group in the amygdala (p_FWE_=.512), hippocampus (p_FWE_=.347) and dACC (p_FWE_=.523). Moreover, there was no significant baseline activity difference between the non-responder and HC group in the amygdala (p_FWE_=.422), hippocampus (p_FWE_=.375) and dACC (p_FWE_=.618).

# Supplement 10: Robustness checks of functional activity analyses

## Methods

To evaluate the robustness of the findings regarding the effects of CBT on brain function, several robustness checks were performed. To verify that our results were not affected by non-linear age effects or depressive symptom severity at baseline (t_0_), the 2x2 ANOVA with group (patient vs. HC group) as a between-subjects factor and time (t_0_ vs. t_1_) as a within-subjects factor was recalculated including age^2^ or HDRS score at baseline as a further covariate (besides age and sex). The subsequent 2x2 ANOVA with response (responders vs. non-responders) as a between-subjects factor and the correlation analyses were also repeated with the respective covariate included.

Moreover, to ensure that the results of the responders vs. non-responders analyses were not driven by clinical differences between the subgroups, these analyses underwent additional sensitivity checks and were repeated while correcting for several clinical characteristics. The number of depressive episodes before study participation (baseline/t_0_) as well as the remission status of the depressive disorder at baseline was collected at baseline using self-reports and validated by the SCID-I (4). Furthermore, the presence of acute comorbidities was assessed during the SCID-I (4). At both time points, information about the current medication intake was collected. To consider psychopharmacological treatment, a medication load index was computed for each time point according to the procedure described by Hassel et al. (11). Each medication was coded as absent = 0, low = 1 (equal or lower average dose), or high = 2 (greater than average dose) in relation to the midpoint of the daily dose range recommended by the Physician’s-Desk-Reference (12). Then, for each participant and time point, a composite measure of total medication exposure was calculated by summing all individual medication. These four variables (number of depressive episodes before baseline, remission status at baseline (no/ partial/ full remission), acute comorbidity (no/ yes) and medication load index) were added as further covariates, on top of age and sex, to the 2x2 ANOVA with response (responders vs. non-responders) as a between-subjects factor and time (t_0_ vs. t_1_) as a within-subjects factor.

## Results

When controlling for age^2^ and depressive symptom severity at baseline, the results of the 2x2 ANOVAs remained significant (**Table S6**). Briefly, there was still a significant group (patient vs. HC group) x time (t_0_ vs. t_1_) interaction within the right hippocampus (both p_FWE_≤.023), resulting from significant bilateral activity decreases within the patient group (all p_FWE_≤.027). Moreover, the main effect of time in the right dACC remained significant when controlling for age^2^ (p_FWE_=.048) as well as for HDRS scores at baseline (p_FWE_=.047), marked by a significant decrease in dACC activity within the patient group (both p_FWE_≤.027). There was still no significant interaction effect or main effect of time in the amygdala (both p_FWE_≥.191) when controlling for age^2^ or HDRS score at baseline. Moreover, the subsequent analyses of responders vs. non-responders still only showed nominally significant response x time interaction effects within the hippocampus when adding the respective covariate (both p_FWE_≤.069). Post hoc *t*-tests also remained significant revealing bilateral hippocampal activity decreases during negative emotion processing in responders only (all p_FWE_≤.049). The main effect of time within the right dACC in the responders vs. non-responders analysis also remained significant (both p_FWE_≤.030), with no significant results observed in the post hoc *t*-tests. The pre-treatment activity difference within the right hippocampus between responders and non-responders also showed continued statistical significance (both p_FWE_≤.018).

Regarding the correlation analyses, when additionally controlling for age^2^, the association between decreases in left hippocampal activity from t_0_ to t_1_ in the patient group and decreases in HDRS scores showed only a tendency toward significance (Left: r=.242 p=.067; Right: p=.698). In contrast, the association between reduction in left hippocampal activity and ΔHDRS remained significant when controlling for depressive symptom severity at baseline (Left: r=.292 p=.026; Right: p=.867). There was still no significant correlation between reductions in bilateral hippocampal activity and BDI score changes when controlling for age^2^ and depressive symptom severity at baseline (all p≥.114). Moreover, correlation analyses between right dACC activity decreases within the patient group from t_0_ to t_1_ and depressive symptom improvement also remained insignificant (HDRS: both p≥.714; BDI: both p≥.563).

When additionally controlling for clinical characteristics (number of depressive episodes before baseline, remission status at baseline, acute comorbidity and medication load index) in the 2x2 ANOVA with response (responders vs. non-responders) as a between-subjects factor, the findings remained significant (**Table S7**). A significant interaction was even observed within the right hippocampus (both p_FWE_≤.035), driven by significant activity decreases in the responder group only (all p_FWE_≤.027). The main effect of time within the right dACC in the responders vs. non-responders analysis also remained significant (both p_FWE_=.018), with no significant results in the post hoc *t*-tests. Additionally, the pre-treatment activity difference within the right hippocampus between responders and non-responders remained statistically significant (p_FWE_=.019).

# Supplement 11: Effect of CBT on whole-brain activity

Whole-brain analysis revealed significant activity decreases in regions such as the middle temporal gyrus and precuneus in the patient group after CBT. The middle temporal gyrus is implicated in social cognition (13), where decreased activity may reflect improved processing of social information, such as negative emotional facial expressions. The precuneus, a part of the default mode network linked to self-referential processing and rumination (14,15), shows reduced activity that may indicate less engagement in these processes post-CBT. Additionally, the whole-brain finding of hippocampal activity decreases aligns with our ROI results. However, no significant decreases were found in either responders or non-responders at the whole-brain level.

# Supplementary Tables

| **Table S1.** Details on prior treatments in the patient group | | | | |
| --- | --- | --- | --- | --- |
|  | Patient group  *n*=59 | Responders  *n*=40 | Non-responders  *n*=19 | *p*-value ^a^ |
|  | Mean (SD) | Mean (SD) | Mean (SD) |  |
| **Psychotherapeutic treatment** | | | | |
| Number of psychotherapeutic treatment trials before t_0_ (no information/0/1/ 2), number of patients ^b^ | 4/37/13/5 | 3/26/8/3 | 1/11/5/2 | .792 |
| Cognitive-behavioral therapy | 10 | 8 | 2 | - |
| Psychoanalytic psychotherapy | 2 | 0 | 2 | - |
| Psychodynamic psychotherapy | 7 | 2 | 5 | - |
| Other method | 0 | 0 | 0 | - |
| No information regarding method | 4 | 4 | 0 | - |
| **Psychopharmacological treatment** | | | | |
| Number of psychopharmacological treatment trials before t_0_ (no information/ 0/1/2/3), number of patients ^b^ | 1/42/10/4/2 | 0/29/6/3/2 | 1/13/4/1/0 | .484 |
| NaSSA | 3 | 3 | 0 | - |
| Tricyclics | 1 | 0 | 1 | - |
| NDRI | 1 | 1 | 0 | - |
| SSRI | 9 | 5 | 4 | - |
| SNRI | 2 | 2 | 0 | - |
| MAO-Inhibitors | 0 | 0 | 0 | - |
| Antipsychotics | 2 | 2 | 0 | - |
| Other | 6 | 5 | 1 | - |
| Number of patients under psycho-pharmacological medication at t_0_ (no/yes) ^b^ | 40/19 | 25/15 | 15/4 | .206 |
| Psychopharmacological medication at t_0_, number of patients ^c^ | | | | |
| NaSSA | 5 | 3 | 2 | - |
| Tricyclics | 0 | 0 | 0 | - |
| NDRI | 1 | 1 | 0 | - |
| SSRI | 10 | 9 | 1 | - |
| SNRI | 5 | 2 | 3 | - |
| MAO-Inhibitors | 0 | 0 | 0 | - |
| Antipsychotics | 2 | 2 | 0 | - |
| Other | 0 | 0 | 0 | - |
| Number of patients under psycho-pharmacological medication at t_1_ (no/yes) ^b^ | 35/24 | 24/16 | 11/8 | .878 |
| Psychopharmacological medication at t_1_, number of patients ^c^ | | | | |
| NaSSA | 3 | 1 | 2 | - |
| Tricyclics | 0 | 0 | 0 | - |
| NDRI | 1 | 1 | 0 | - |
| SSRI | 14 | 9 | 5 | - |
| SNRI | 6 | 3 | 3 | - |
| MAO-Inhibitors | 0 | 0 | 0 | - |
| Antipsychotics | 2 | 0 | 2 | - |
| Other | 1 | 0 | 1 | - |
| Medication load index at t_0_ | .53 (.88) | .55 (.82) | .47 (1.02) | .758 |
| Medication load index at t_1_ | .59 (.95) | .43 (.68) | .95 (1.31) | .116 |
| Δ Medication load index t_0_ – t_1_ | -.068 (.89) | .13 (.85) | -.47 (.84) | Patient group: .560  Responder: .360  Non-responder: .025 |
| NaSSA, noradrenergic and specific serotonergic antidepressant; NDRI, norepinephrine and dopamine reuptake inhibitors; SSRI, selective serotonin reuptake inhibitors; SNRI, serotonin and norepinephrine uptake inhibitors; MAO-Inhibitors, monoamine oxidase inhibitors.  ^a^ Comparing patients from the responder and non-responder group by using the unpaired two-tailed *t*-test except where noted.  ^b^ Comparing patients from the responder and non-responder group by using the χ^2^-test.  ^c^ Information of whether at least one substance from this drug class is being taken at the specified assessment time point. | | | | |

| **Table S2**. Results of the group x time ANOVAs in all three regions of interest | | | | | | | | | |
| --- | --- | --- | --- | --- | --- | --- | --- | --- | --- |
| Region of interest | Side | Cluster size | Peak voxel coordinates | | | *TFCE-*value | *T*- value | *p_FWE_*- value | *η_p_²* [95.%-CI] |
|  |  |  | x | y | z |  |  |  |  |
| **Hippocampus** | | | | | | | | | |
| **Group (patient vs. HC group) x time (t_0_ vs. t_1_)** | | | | | | | | | |

| *Interaction effect ^1^* | R | 26 | 24 | -40 | 6 | 120.33 | 4.10 | **.022** | .101  [.0210, .2100] |
| --- | --- | --- | --- | --- | --- | --- | --- | --- | --- |
| *Main effect of time ^1^* | R | 17 | 36 | -28 | -6 | 118.64 | 4.04 | **.028** | .124  [.0334, .2372] |
| *Post-hoc tests from t_0_ to t_1_ ^2^* | | | | | | | | | |
| Patient group: t_0_ > t_1_ | R | 34 | 26 | -34 | 8 | 130.48 | 3.93 | **.022** | .185  [.0383, .3497] |
|  | L | 9 | -16 | -34 | 10 | 127.32 | 4.48 | **.024** | .204  [.0489, .3686] |
|  | R | 3 | 36 | -36 | -4 | 101.11 | 3.27 | **.046** | .111  [.0071, .2695] |
| HC group: t_0_ < t_1_ | - | - | - | - | - | - | - | .420 | - |
| t_0_: patient group > HC group | - | - | - | - | - | - | - | .232 | - |
| t_1_: patient group < HC group | - | - | - | - | - | - | - | .283 | - |
| **Group (responders vs. non-responders) x time (t_0_ vs. t_1_)** | | | | | | | | | |
| *Interaction effect ^3^* | - | - | - | - | - | - | - | .068 | - |
| *Main effect of time ^3^* | - | - | - | - | - | - | - | .087 | - |
| *Post-hoc tests from from t_0_ to t_1_ ^4^* | | | | | | | | | |
| Responder group: t_0_ > t_1_ | R | 53 | 18 | -32 | 10 | 219.51 | 4.82 | **.005** | .260  [.0556, .4509] |
|  | L | 73 | -34 | -34 | -2 | 155.21 | 3.93 | **.020** | .204  [.0269, .3999] |
|  | R | 21 | 34 | -36 | 0 | 134.82 | 3.70 | **.030** | .220  [.0341, .4147] |
|  | R | 18 | 38 | -24 | -8 | 132.16 | 3.76 | **.032** | .249  [.0497, .4417] |
|  | L | 7 | -16 | -34 | 10 | 121.66 | 4.16 | **.040** | .233  [.0407, .4267] |
| Non-responder group:  t_0_ < t_1_ | - | - | - | - | - | - | - | .220 | - |
| t_0_: responders > non-responders ^4^ | R | 44 | 34 | -18 | -14 | 127.07 | 3.73 | .**017** | .189  [.0397, .3554] |
| t_1_: responders < non-responders ^4^ | - | - | - | - | - | - | - | .709 | - |
| **Amygdala** | | | | | | | | | |
| **Group (patient vs. HC group) x time (t_0_ vs. t_1_)** | | | | | | | | | |
| *Interaction effect ^1^* | - | - | - | - | - | - | - | .198 | - |
| *Main effect of time ^1^* | - | - | - | - | - | - | - | .198 | - |
| t_0_: patient group > HC group | - | - | - | - | - | - | - | .497 | - |
| t_1_: patient group > HC group | - | - | - | - | - | - | - | .125 | - |
| **Group (responders vs. non-responders) x time (t_0_ vs. t_1_)** | | | | | | | | | |
| *Interaction effect ^3^* | - | - | - | - | - | - | - | .522 | - |
| *Main effect of time ^3^* | - | - | - | - | - | - | - | .416 | - |
| t_0_: responders > non-responders ^4^ | - | - | - | - | - | - | - | .267 | - |
| t_1_: responders > non-responders ^4^ | - | - | - | - | - | - | - | .242 | - |
| **Dorsal anterior cingulate cortex** | | | | | | | | | |
| **Group (patient vs. HC group) x time (t_0_ vs. t_1_)** | | | | | | | | | |
| *Interaction effect ^1^* | - | - | - | - | - | - | - | .173 | - |
| *Main effect of time ^1^* | R | 5 | 20 | 36 | 22 | 134.22 | 3.94 | **.043** | .098  [.0195, .2063] |
| *Post-hoc tests from t_0_ to t_1_ ^2^* | | | | | | | | | |
| Patient group: t_0_ > t_1_ | R | 11 | 20 | 36 | 22 | 174.60 | 4.94 | **.022** | .233  [.0669, .3965] |
| HC group: t_0_ > t_1_ | - | - | - | - | - | - | - | .120 | - |
| t_0_: patient group > HC group | - | - | - | - | - | - | - | .608 | - |
| t_1_: patient group < HC group | - | - | - | - | - | - | - | .424 | - |
| **Group (responders vs. non-responders) x time (t_0_ vs. t_1_)** | | | | | | | | | |
| *Interaction effect ^3^* | - | - | - | - | - | - | - | .276 | - |
| *Main effect of time ^3^* | R | 19 | 20 | 36 | 22 | 177.33 | 4.90 | **.018** | .219  [.0584, .3839] |
| *Post-hoc tests from from t_0_ to t_1_ ^4^* | | | | | | | | | |
| Responder group: t_0_ > t_1_ | - | - | - | - | - | - | - | .179 | - |
| Non-responder group:  t_0_ > t_1_ | - | - | - | - | - | - | - | .092 | - |
| t_0_: responders < non-responders ^4^ | - | - | - | - | - | - | - | .371 | - |
| t_1_: responders > non-responders ^4^ | - | - | - | - | - | - | - | .414 | - |

| HC, healthy controls. T_0_, before the start of cognitive behavioural therapy. T_1_, after approximately 20 sessions of cognitive behavioural therapy.  ^1^ *df_1_*= 1; *df_2_*=232.  ^2^ *df*=232.  ^3^ *df_1_*= 1; *df_2_*=112.  ^4^ *df*=112. |
| --- |

| **Table S3**. Results of the main effect of time at whole-brain level | | | | | | | | | |
| --- | --- | --- | --- | --- | --- | --- | --- | --- | --- |
| Anatomical region ^1^ | Side | Cluster size ^2^ | Peak voxel coordinates | | | *TFCE-*value | *T*- value | *p_FWE_*- value | *η_p_²* [95.%-CI] |
|  |  |  | x | y | z |  |  |  |  |
| **Group (patient vs. HC) x time (t_0_ vs. t_1_)** | | | | | | | | |  |

| *Main effect of time ^3^* |  |  |  |  |  |  |  |  |  |
| --- | --- | --- | --- | --- | --- | --- | --- | --- | --- |
| Precuneus/ angular gyrus/ middle frontal gyrus/ superior frontal gyrus/ caudate nucleus/ middle temporal gyrus | L | 11854 | -12 | 12 | 16 | 1312.26 | 4.95 | **.007** | .145  [.0468, .2611] |
| Inferior frontal gyrus | L | 128 | -52 | 26 | 8 | 905.76 | 4.15 | **.036** | .125  [.0346, .2386] |
| Superior frontal gyrus/ middle frontal gyrus/ supplementary motor area | R | 334 | 16 | 30 | 58 | 884.47 | 3.49 | **.040** | .105  [.0232, .2143] |
| *Post-hoc tests from from t_0_ to t_1_ ^4^* | | | | | | | | |  |
| Patient group: t_0_ > t_1_ |  |  |  |  |  |  |  |  |  |
| Precuneus/ middle temporal gyrus/ lingual gyrus | L | 2263 | -14 | -36 | 12 | 1122.67 | 4.67 | **.019** | .240  [.0720, .4038] |
| Caudate nucleus | L | 302 | -20 | 20 | 16 | 1095.79 | 5.09 | **.021** | .250  [.0788, .4130] |
| Caudate nucleus | R | 486 | 20 | 26 | 16 | 1022.79 | 4.65 | **.028** | .210  [.0526, .3746] |
| Precuneus/ hippocampus | R | 736 | 26 | -34 | 8 | 992.33 | 3.93 | **.031** | .279  [.1002, .4400] |
| Caudate nucleus | L | 260 | -20 | 32 | 6 | 975.86 | 4.23 | **.033** | .230  [.0650, .3938] |
| HC group: t_0_ > t_1_ | - | - | - | - | - | - | - | .159 | - |

| HC, healthy controls. T_0_, before the start of cognitive behavioural therapy. T_1_, after approximately 20 sessions of cognitive behavioural therapy.  ^1^ Only the regions with at least 4% participation in the significant cluster are reported.  ^2^ Only significant clusters (p_FWE_ <.05) with cluster size k>100 are reported.  *^3^ df_1_*= 1; *df_2_*=232.  ^4^ *df*=232. |
| --- |

| **Table S4**. Best hyperparameter configurations of the Random Forest Classifier | | | | | |
| --- | --- | --- | --- | --- | --- |
| Classification model | Anatomical region | Max_ features | Min_samples_leaf | Min_samples_split | N_ estimators |
| t_0_: responders vs. non-responders | Hippocampus | sqrt | 0.1 | 3 | 7 |
|  | Amygdala | sqrt | 0.01 | 3 | 6 |
|  | dACC | sqrt | 0.01 | 3 | 5 |
|  | Whole-brain | log2 | 0.01 | 3 | 8 |
| T_0_, before the start of cognitive behavioural therapy. T_1_, after 20 sessions of cognitive behavioural therapy. dACC, dorsal anterior cingulate cortex. Max_features, maximum number of features considered for splitting a node. Min_samples_leaf, minimun number of data points allowed in a leaf node. Min_samples_split, minimum number of data points placed in a node before the node is split. N_estimators, number of trees in the foreset. | | | | | |

| **Table S5**. Detailed results of the classification accuracy based on the ROI and whole-brain results (mean and standard deviation) | | | | | | | |
| --- | --- | --- | --- | --- | --- | --- | --- |
| Anatomical region | Classification Model | BACC | ACC | Sensitivity | Specificity | F1 score | n |
| t_0_: responders vs. non-responders | Hippocampus | 0.483 (0.102) | 0.596 (0.117) | 0.800 (0.150) | 0.167 (0.139) | 0.723 (0.097) | 40/19 |
|  | Amygdala | 0.479 (0.108) | 0.577 (0.069) | 0.775 (0.094) | 0.183 (0.260) | 0.713 (0.043) | 40/19 |
|  | dACC | 0.604 (0.108) | 0.680 (0.119) | 0.825 (0.170) | 0.383 (0.172) | 0.770 (0.103) | 40/19 |
|  | Whole-Brain | 0.479 (0.152) | 0.562 (0.165) | 0.725 (0.242) | 0.233 (0.244) | 0.672 (0.184) | 40/19 |
| ROI, region of interest. BACC, balanced accuracy. ACC, accuracy. T_0_, before the start of cognitive behavioural therapy. T_1_, after 20 sessions of cognitive behavioural therapy. dACC, dorsal anterior cingulate cortex. | | | | | | | |

| **Table S6**. Results of the group x time ANOVAs in all three regions of interest with age^2^ or HDRS score at baseline as additional covariates (including age and sex) | | | | | | | | |
| --- | --- | --- | --- | --- | --- | --- | --- | --- |
| Region of interest | Side | Cluster size | Peak voxel coordinates | | | *TFCE-*value | *T*- value | *p_FWE_*- value |
|  |  |  | x | y | z |  |  |  |
| **Hippocampus** | | | | | | | | |
| *Controlling for age^2^* | | | | | | | | |
| **Group (patient vs. HC group) x time (t_0_ vs. t_1_)** | | | | | | | | |

| *Interaction effect ^1^* | R | 20 | 24 | -40 | 6 | 114.57 | 4.09 | **.023** |
| --- | --- | --- | --- | --- | --- | --- | --- | --- |
| *Main effect of time ^1^* | R | 17 | 36 | -28 | -6 | 114.03 | 4.07 | **.028** |
| *Post-hoc tests from t_0_ to t_1_ ^2^* | | | | | | | | |
| Patient group: t_0_ > t_1_ | R | 20 | 26 | -34 | 8 | 122.45 | 3.09 | **.025** |
|  | L | 7 | -16 | -34 | 10 | 119.20 | 4.46 | **.027** |
| HC group: t_0_ < t_1_ | - | - | - | - | - | - | - | .414 |
| t_0_: patient group > HC group | - | - | - | - | - | - | - | .161 |
| t_1_: patient group < HC group | - | - | - | - | - | - | - | .359 |
| **Group (responders vs. non-responders) x time (t_0_ vs. t_1_)** | | | | | | | | |
| *Interaction effect ^3^* | - | - | - | - | - | - | - | .069 |
| *Main effect of time ^3^* | - | - | - | - | - | - | - | .104 |
| *Post-hoc tests from from t_0_ to t_1_ ^4^* | | | | | | | | |
| Responder group: t_0_ > t_1_ | R | 47 | 18 | -32 | 10 | 203.68 | 4.75 | **.006** |
|  | L | 61 | -34 | -34 | -2 | 145.14 | 3.87 | **.023** |
|  | R | 10 | 34 | -36 | 0 | 122.33 | 3.64 | **.038** |
|  | R | 6 | 38 | -24 | -8 | 117.39 | 3.69 | **.042** |
|  | L | 2 | -16 | -34 | 10 | 112.96 | 4.13 | **.047** |
|  | L | 1 | -12 | -36 | 10 | 111.87 | 3.31 | **.048** |
|  | L | 1 | -36 | -30 | -12 | 110.36 | 2.92 | **.049** |
| Non-responder group:  t_0_ < t_1_ | - | - | - | - | - | - | - | .201 |
| t_0_: responders > non-responders ^4^ | R | 60 | 36 | -18 | -14 | 133.10 | 3.76 | .014 |
| t_1_: responders < non-responders ^4^ | - | - | - | - | - | - | - | .774 |
| *Controlling for depressive symptom severity at baseline* | | | | | | | | |
| **Group (patient vs. HC group) x time (t0 vs. t1)** | | | | | | | | |
| *Interaction effect ^1^* | R | 25 | 24 | -40 | 6 | 118.91 | 4.11 | **.022** |
| *Main effect of time ^1^* | R | 17 | 36 | -28 | -6 | 115.70 | 4.05 | **.029** |
| *Post-hoc tests from t_0_ to t_1_ ^2^* | | | | | | | | |
| Patient group: t_0_ > t_1_ | R | 53 | 26 | -34 | 8 | 129.40 | 3.94 | **.018** |
|  | L | 12 | -16 | 34 | 10 | 126.42 | 1.77 | **.020** |
| HC group: t_0_ < t_1_ | - | - | - | - | - | - | - | .420 |
| t_0_: patient group > HC group | - | - | - | - | - | - | - | .263 |
| t_1_: patient group < HC group | - | - | - | - | - | - | - | .256 |
| **Group (responders vs. non-responders) x time (t0 vs. t1)** | | | | | | | | |
| *Interaction effect ^3^* | - | - | - | - | - | - | - | .068 |
| *Main effect of time ^3^* | - | - | - | - | - | - | - | .086 |
| *Post-hoc tests from from t_0_ to t_1_ ^4^* | | | | | | | | |
| Responder group: t_0_ > t_1_ | R | 53 | 18 | -32 | 10 | 218.40 | 4.83 | **.005** |
|  | L | 73 | -34 | -34 | -2 | 154.56 | 3.93 | **.020** |
|  | R | 21 | 34 | -36 | 0 | 134.17 | 3.70 | **.031** |
|  | R | 13 | 38 | -24 | -8 | 127.97 | 3.76 | **.035** |
|  | L | 6 | -16 | -34 | 10 | 122.22 | 4.16 | **.040** |
| Non-responder group:  t_0_ < t_1_ | - | - | - | - | - | - | - | .216 |
| t_0_: responders > non-responders ^4^ | R | 42 | 34 | -18 | -14 | 123.37 | 3.71 | **.018** |
| t1: responders < non-responders 4 | - | - | - | - | - | - | - | .719 |
| **Amygdala** | | | | | | | | |
| *Controlling for age^2^* | | | | | | | | |
| **Group (patient vs. HC group) x time (t_0_ vs. t_1_)** | | | | | | | | |
| *Interaction effect ^1^* | - | - | - | - | - | - | - | .191 |
| *Main effect of time ^1^* | - | - | - | - | - | - | - | .258 |
| t_0_: patient group > HC group | - | - | - | - | - | - | - | .525 |
| t_1_: patient group > HC group | - | - | - | - | - | - | - | .118 |
| **Group (responders vs. non-responders) x time (t_0_ vs. t_1_)** | | | | | | | | |
| *Interaction effect ^3^* | - | - | - | - | - | - | - | .525 |
| *Main effect of time ^3^* | - | - | - | - | - | - | - | .397 |
| t_0_: responders > non-responders ^4^ | - | - | - | - | - | - | - | .251 |
| t_1_: responders > non-responders ^4^ | - | - | - | - | - | - | - | .238 |
| *Controlling for depressive symptom severity at baseline* | | | | | | | | |
| **Group (patient vs. HC group) x time (t0 vs. t1)** | | | | | | | | |
| *Interaction effect ^1^* | - | - | - | - | - | - | - | .196 |
| *Main effect of time ^1^* | - | - | - | - | - | - | - | .246 |
| t_0_: patient group > HC group | - | - | - | - | - | - | - | .327 |
| t_1_: patient group > HC group | L | 33 | -24 | -4 | -14 | 47.55 | 3.06 | **.017** |
| **Group (responders vs. non-responders) x time (t0 vs. t1)** | | | | | | | | |
| *Interaction effect ^3^* | - | - | - | - | - | - | - | .519 |
| *Main effect of time ^3^* | - | - | - | - | - | - | - | .415 |
| t_0_: responders > non-responders ^4^ | - | - | - | - | - | - | - | .270 |
| t_1_: responders > non-responders ^4^ | - | - | - | - | - | - | - | .248 |
| **Dorsal anterior cingulate cortex** | | | | | | | | |
| *Controlling for age^2^* | | | | | | | | |
| **Group (patient vs. HC group) x time (t_0_ vs. t_1_)** | | | | | | | | |
| *Interaction effect ^1^* | - | - | - | - | - | - | - | .185 |
| *Main effect of time ^1^* | R | 1 | 20 | 36 | 22 | 123.59 | 3.92 | **.048** |
| *Post-hoc tests from t_0_ to t_1_ ^2^* | | | | | | | | |
| Patient group: t_0_ > t_1_ | R | 10 | 20 | 36 | 22 | 160.12 | 4.92 | **.027** |
| HC group: t_0_ > t_1_ | - | - | - | - | - | - | - | .121 |
| t_0_: patient group > HC group | - | - | - | - | - | - | - | .572 |
| t_1_: patient group < HC group | - | - | - | - | - | - | - | .453 |
| **Group (responders vs. non-responders) x time (t_0_ vs. t_1_)** | | | | | | | | |
| *Interaction effect ^3^* | - | - | - | - | - | - | - | .267 |
| *Main effect of time ^3^* | R | 10 | 20 | 36 | 22 | 147.58 | 4.77 | **.030** |
| *Post-hoc tests from from t_0_ to t_1_ ^4^* | | | | | | | | |
| Responder group: t_0_ > t_1_ | - | - | - | - | - | - | - | .225 |
| Non-responder group: t_0_ > t_1_ | - | - | - | - | - | - | - | .116 |
| t_0_: responders < non-responders ^4^ | - | - | - | - | - | - | - | .507 |
| t_1_: responders > non-responders ^4^ | - | - | - | - | - | - | - | .378 |
| *Controlling for depressive symptom severity at baseline* | | | | | | | | |
| **Group (patient vs. HC group) x time (t_0_ vs. t_1_)** | | | | | | | | |
| *Interaction effect ^1^* | - | - | - | - | - | - | - | .186 |
| *Main effect of time ^1^* | R | 3 | 20 | 36 | 22 | 127.91 | 3.94 | **.047** |
| *Post-hoc tests from t_0_ to t_1_ ^2^* | | | | | | | | |
| Patient group: t_0_ > t_1_ | R | 10 | 20 | 36 | 22 | 163.73 | 4.95 | **.026** |
| HC group: t_0_ > t_1_ | - | - | - | - | - | - | - | .117 |
| t_0_: patient group > HC group | - | - | - | - | - | - | - | .485 |
| t_1_: patient group < HC group | - | - | - | - | - | - | - | >.999 |
| **Group (responders vs. non-responders) x time (t_0_ vs. t_1_)** | | | | | | | | |
| *Interaction effect ^3^* | - | - | - | - | - | - | - | .273 |
| *Main effect of time ^3^* | R | 12 | 20 | 36 | 22 | 162.21 | 4.89 | **.023** |
| *Post-hoc tests from from t_0_ to t_1_ ^4^* | | | | | | | | |
| Responder group: t_0_ > t_1_ | - | - | - | - | - | - | - | .192 |
| Non-responder group: t_0_ > t_1_ | - | - | - | - | - | - | - | .102 |
| t_0_: responders < non-responders ^4^ | - | - | - | - | - | - | - | .373 |
| t_1_: responders > non-responders ^4^ | - | - | - | - | - | - | - | .419 |
| HC, healthy controls. T_0_, before the start of cognitive behavioural therapy. T_1_, after approximately 20 sessions of cognitive behavioural therapy.  ^1^ *df_1_*= 1; *df_2_*=231.  ^2^ *df*=231.  ^3^ *df_1_*= 1; *df_2_*=111.  ^4^ *df*=111. | | | | | | | | |

| **Table S7**. Results of the response x time ANOVAs in all three regions of interest with medication load index, acute comorbidity (no/ yes), remission status at baseline (no/ partial/ full remission) and number of depressive episodes before baseline, on top of age and sex, as covariates. | | | | | | | | |
| --- | --- | --- | --- | --- | --- | --- | --- | --- |
| Region of interest | Side | Cluster size | Peak voxel coordinates | | | *TFCE-*value | *T*- value | *p_FWE_*- value |
|  |  |  | x | y | z |  |  |  |
| **Hippocampus** | | | | | | | | |

| **Group (responders vs. non-responders) x time (t_0_ vs. t_1_)** | | | | | | | | |
| --- | --- | --- | --- | --- | --- | --- | --- | --- |
| *Interaction effect ^1^* | R | 21 | 34 | -20 | -12 | 122.07 | 3.92 | **.023** |
|  | R | 5 | 16 | -32 | 10 | 105.97 | 4.58 | **.035** |
| *Main effect of time ^1^* | - | - | - | - | - | - | - | .082 |
| *Post-hoc tests from from t_0_ to t_1_ ^2^* | | | | | | | | |
| Responder group: t_0_ > t_1_ | R | 158 | 18 | -32 | 10 | 248.50 | 5.12 | **.003** |
|  | L | 14 | -16 | -34 | 10 | 140.81 | 4.37 | **.026** |
|  | L | 51 | -34 | -34 | -2 | 138.54 | 3.75 | **.027** |
| Non-responder group:  t_0_ < t_1_ | - | - | - | - | - | - | - | .136 |
| t_0_: responders > non-responders ^2^ | R | 45 | 36 | -18 | -14 | 129.39 | 3.64 | **.019** |
| t_1_: responders < non-responders ^2^ | - | - | - | - | - | - | - | .729 |
| **Amygdala** | | | | | | | | |
| **Group (responders vs. non-responders) x time (t_0_ vs. t_1_)** | | | | | | | | |
| *Interaction effect ^1^* | - | - | - | - | - | - | - | .511 |
| *Main effect of time ^1^* | - | - | - | - | - | - | - | .509 |
| t_0_: responders > non-responders ^2^ | - | - | - | - | - | - | - | .320 |
| t_1_: responders > non-responders ^2^ | - | - | - | - | - | - | - | .405 |
| **Dorsal anterior cingulate cortex** | | | | | | | | |
| **Group (responders vs. non-responders) x time (t_0_ vs. t_1_)** | | | | | | | | |
| *Interaction effect ^1^* | - | - | - | - | - | - | - | .228 |
| *Main effect of time ^1^* | R | 21 | 18 | 36 | 22 | 175.48 |  | **.018** |
| *Post-hoc tests from from t_0_ to t_1_ ^2^* | | | | | | | | |
| Responder group: t_0_ > t_1_ | - | - | - | - | - | - | - | .222 |
| Non-responder group: t_0_ > t_1_ | - | - | - | - | - | - | - | .080 |
| t_0_: responders < non-responders ^2^ | - | - | - | - | - | - | - | .412 |
| t_1_: responders > non-responders ^2^ | - | - | - | - | - | - | - | .482 |
| T_0_, before the start of cognitive behavioural therapy. T_1_, after approximately 20 sessions of cognitive behavioural therapy.  ^1^ *df_1_*= 1; *df_2_*=108.  ^2^ *df*=108. | | | | | | | | |

# Supplementary Figures

## **Figure S1.** Flow diagram visualizing the participant dropout process

Participants included in the PINC study at baseline

*n* = 129 patients, *n* = 119 HC

- Start of a psychotherapeutic treatments other than CBT after baseline: *n* = 7 patients
- Met criteria for a mental disorder at baseline: *n* = 8 HC
- Met criteria for a somatic condition contraindicating our assessments: *n* = 3 HC
- Lost to follow-up: *n* = 22 patients, *n* = 17 HC
- No completion of follow-up assessments by data analysis start: *n* = 4 patients, *n* = 18 HC

Participants included in the PINC study at baseline and follow-up

*n* = 96 patients, *n* = 73 HC

Participants with complete fMRI data at baseline and follow-up

*n* = 69 patients, *n* = 69 HC

Participants with complete fMRI data at baseline and follow-up meeting inclusion criteria

*n* = 59 patients, *n* = 60 HC

- Absence of CBT or other psychotherapeutic treatment between baseline and follow-up: *n* = 19 patients
- Unavailable fMRI images at baseline, follow-up or both: *n* = 8 patients, *n* = 4 HC
- Met criteria for a mental disorder during study interval: *n* = 5 HC
- Met criteria for a fully remitted MDD or partially remitted adjustment disorder at baseline: *n* = 3 patients
- Completion of more than 30 CBT sessions: *n* = 2 patients
- Scored below the BDI cut-off (patients) or above the BDI cut-off (HC): *n* = 5 patients, *n* = 4 HC

**Figure S1. Flow diagram visualizing the participant dropout process.** CBT = Cognitive-behavioral therapy, FMRI = Functional magnetic resonance imaging, HC = Healthy control, MDD = Major depressive disorder, PINC = Prevention and intervention neuroimaging cohort. ^a^ Patients were excluded if they scored below the cut-off for clinically remarkable depressive symptoms (5) on the BDI (6) at baseline, while HC were excluded if they scored above the cut-off at baseline.

## **Figure S2.** Example trial of fMRI paradigm.


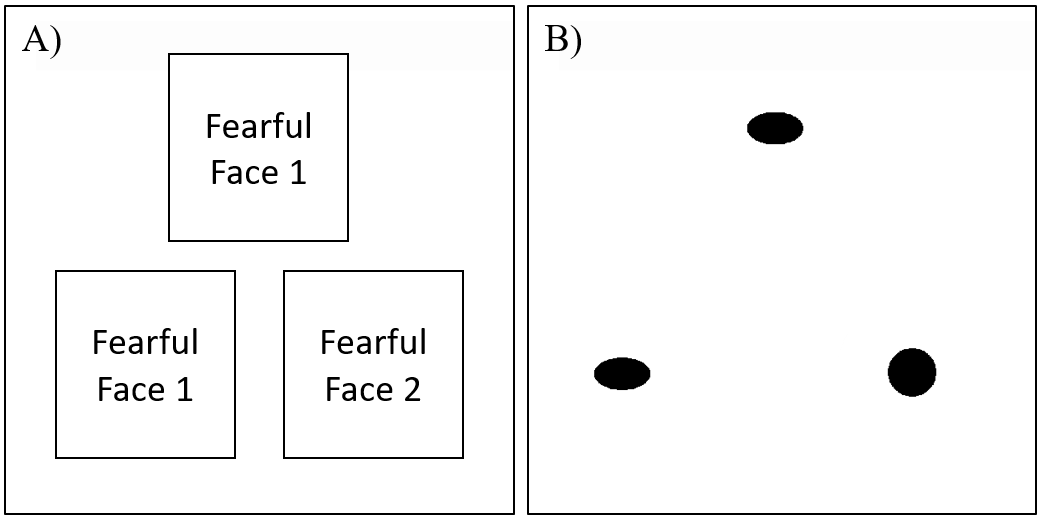


**Figure S2. Example trial of fMRI paradigm. (A)** During each block of the face-processing task, participants were presented trials with a trio of faces (all expressing either fear or anger) and were instructed to match one of two faces at the bottom of the screen that matched the target face at the top via keystroke. **(B)** The sensorimotor control task showed trials with a trio of geometric shapes (circles and ellipses) in each block, following the matching procedure of the face-processing task.

## **Figure S3**. Classification results for responders versus non-responders at baseline


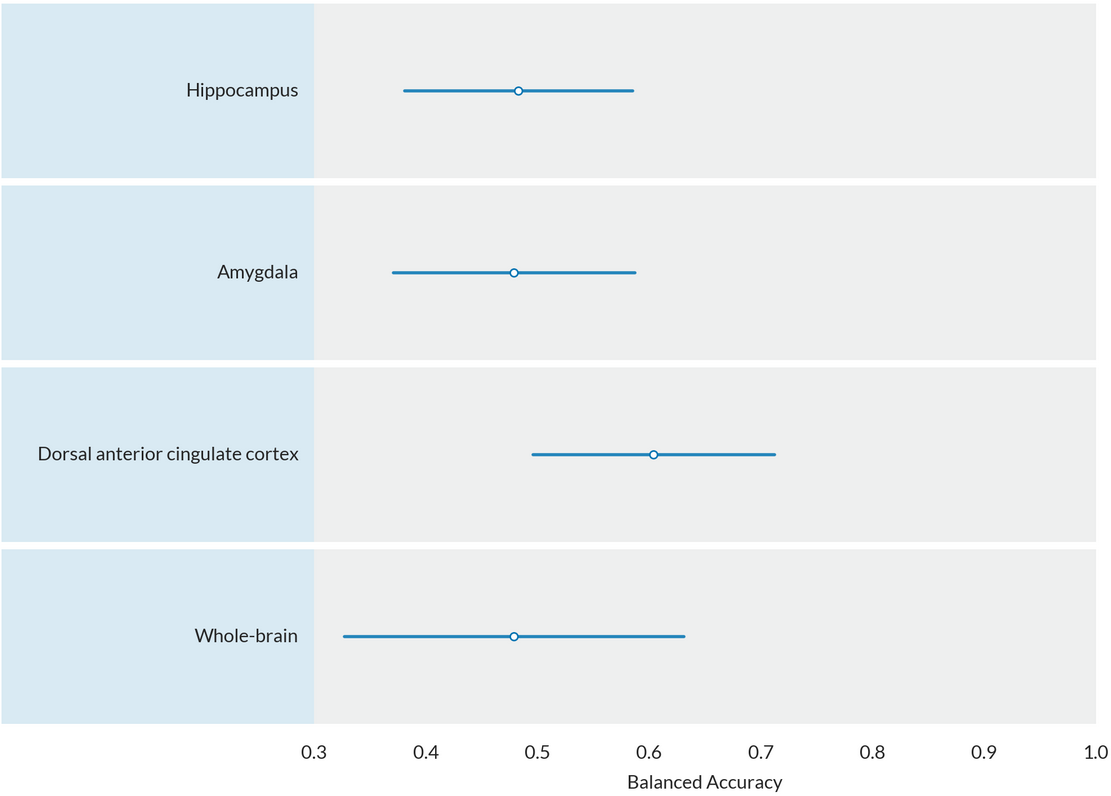


**Figure S3. Classification results for responders versus non-responders.** Balanced accuracy for the best machine learning pipeline in each ROI and at whole-brain for testing the prediction of response group, with error bars indicating a range of +-1 standard deviation computed across the 5 outer cross-validation folds. Figure was created using scripts from Winter et al. (10).

# References

1. Bundesärztekammer (BÄK), Kassenärztliche Bundesvereinigung (KBV), Arbeitsgemeinschaft der Wissenschaftlichen Medizinischen Fachgesellschaften (AWMF), Herausgeber. Nationale VersorgungsLeitlinie Unipolare Depression. BÄK, KBV, AWMF; 2022.

2. Hautzinger M. Kognitive Verhaltenstherapie bei Depressionen. Weinheim ; Basel : Beltz; 2013.

3. Hautzinger M. Akute Depression. Hogrefe Verlag GmbH & Company KG; 2010.

4. Wittchen HU, Wunderlich U, Gruschwitz S, Zaudig M. Strukturiertes Klinisches Interview für DSM-IV: SKID. Achse I: Psychische Störungen. Interviewheft und Beurteilungsheft. Eine deutschsprachige, erweiterte Bearbeitung der amerikanischen Originalversion des SKID-I. Göttingen: Hogrefe; 1997.

5. Beck AT, Steer RA, Carbin MG. Psychometric properties of the Beck Depression Inventory: Twenty-five years of evaluation. Clin Psychol Rev. 1. Januar 1988;8(1):77–100.

6. Beck AT, Steer RA. Beck Depression Inventory: Manual. San Antonio, TX: Psychological Corp, Harcourt Brace Jovanovich; 1987.

7. Hariri AR, Mattay VS, Tessitore A, Kolachana B, Fera F, Goldman D, u. a. Serotonin transporter genetic variation and the response of the human amygdala. Science. 2002;297(5580):400–3.

8. Ekman P, Friesen WV. Pictures of Facial Affect. Paolo Alto, CA: Consulting Psychologists Press; 1976.

9. Leenings R, Winter NR, Plagwitz L, Holstein V, Ernsting J, Steenweg J, u. a. PHOTONAI -- A Python API for rapid machine learning model development. PLoS ONE. 2021;16(7):e0254062.

10. Winter NR, Blanke J, Leenings R, Ernsting J, Fisch L, Sarink K, u. a. A systematic evaluation of machine learning–based biomarkers for major depressive disorder. JAMA Psychiatry. 2024;81(4):386–95.

11. Hassel S, Almeida JR, Kerr N, Nau S, Ladouceur CD, Fissell K, u. a. Elevated striatal and decreased dorsolateral prefrontal cortical activity in response to emotional stimuli in euthymic bipolar disorder: no associations with psychotropic medication load. Bipolar Disord. 2008;10(8):916–27.

12. Reynolds CR. Physician’s Desk Reference. In: Reynolds CR, Fletcher-Janzen E, Herausgeber. Encyclopedia of Special Education. John Wiley & Sons, Ltd; 2008.

13. Xu J, Lyu H, Li T, Xu Z, Fu X, Jia F, u. a. Delineating functional segregations of the human middle temporal gyrus with resting-state functional connectivity and coactivation patterns. Hum Brain Mapp. 2019;40(18):5159–71.

14. Butterfield RD, Grad-Freilich M, Silk JS. The role of neural self-referential processes underlying self-concept in adolescent depression: A comprehensive review and proposed neurobehavioral model. Neurosci Biobehav Rev. 1. Juni 2023;149:105183.

15. Jacob Y, Morris LS, Huang KH, Schneider M, Rutter S, Verma G, u. a. Neural correlates of rumination in major depressive disorder: A brain network analysis. NeuroImage Clin. 2020;25:102142.
